# Thermal-Adaptation-Behavior-Based Thermal Sensation Evaluation Model with Surveillance Cameras

**DOI:** 10.3390/s24041219

**Published:** 2024-02-14

**Authors:** Yu Wang, Wenjun Duan, Junqing Li, Dongdong Shen, Peiyong Duan

**Affiliations:** 1 School of Information Science and Engineering, Shandong Normal University, Jinan 250358, China; wangyu52@stu.sdnu.edu.cn (Y.W.); shendongdong@stu.sdnu.edu.cn (D.S.); 2 School of Computer Science and Technology, Shandong Jianzhu University, Jinan 250101, China; 3 Sohool of Computer Science, Liaocheng University, Liaocheng 252000, China; lijunqing@lcu-cs.com

**Keywords:** thermal sensation evaluation, graph convolutional network, building energy saving, surveillance camera, intelligent building

## Abstract

The construction sector is responsible for almost 30% of the world’s total energy consumption, with a significant portion of this energy being used by heating, ventilation and air-conditioning (HVAC) systems to ensure people’s thermal comfort. In practical applications, the conventional approach to HVAC management in buildings typically involves the manual control of temperature setpoints by facility operators. Nevertheless, the implementation of real-time alterations that are based on the thermal comfort levels of humans inside a building has the potential to dramatically improve the energy efficiency of the structure. Therefore, we propose a model for non-intrusive, dynamic inference of occupant thermal comfort based on building indoor surveillance camera data. It is based on a two-stream transformer-augmented adaptive graph convolutional network to identify people’s heat-related adaptive behaviors. The transformer specifically strengthens the original adaptive graph convolution network module, resulting in further improvement to the accuracy of the detection of thermal adaptation behavior. The experiment is conducted on a dataset including 16 distinct temperature adaption behaviors. The findings indicate that the suggested strategy significantly improves the behavior recognition accuracy of the proposed model to 96.56%. The proposed model provides the possibility to realize energy savings and emission reductions in intelligent buildings and dynamic decision making in energy management systems.

## 1. Introduction

Energy use in buildings is a substantial component of worldwide energy consumption [1,2,3]. Conventional building management systems function based on fixed or unchanging operating schedules. Prior research has shown that a considerable amount of energy is squandered in places that are either underutilized or overutilized [4,5]. Furthermore, the presence of individuals and their utilization of equipment have a role in the internal energy consumption and affect the temperature of the environment. The domain of thermal comfort inference has been extensively researched and has garnered significant interest due to its potential to greatly enhance building energy efficiency while ensuring that occupants are satisfied with their interior settings [6,7,8,9]. Thermal sensation detection algorithms, derived from thermal adaption behaviors seen using a camera, are an innovative and noninvasive means of detecting thermal sensations without the need for further detection equipment [10]. This subject has been extensively researched for many years and continues to be popular because of its significant potential in promoting energy saving in construction. Therefore, it is crucial to accurately assess an individual’s temperature perception in order to effectively regulate air-conditioning systems in real time.

Thermal comfort inference is a challenging task. There have been various attempts to evaluate thermal comfort with thermoregulatory systems, wearable sensors and visual imaging equipment. The most widely used thermoregulatory-system-based thermal comfort model [11] predicts the percentage of satisfied occupants based on the predicted mean vote (PMV) and the predicted percentage of dissatisfied (PPD) occupants; this approach is thus known as the PMV-PPD model. This model evaluates the correlations between human thermal sensation and six parameters: namely, the wind speed, temperature, humidity, longwave radiation, amount of clothing and activity. Although the PMV-PPD model has achieved promising results and has been used extensively in thermal comfort quantification [12,13,14,15], there are still significant constraints, such as the lack of precision in determining individual characteristics, which will lead to the underestimation or overestimation of personalized thermal comfort [16,17]. Furthermore, this model ignores a vital factor, which is the physiological traits of individuals [16,17].

The identification of physiological variables is accomplished via the use of invasive physiological monitoring equipment by thermal comfort perception approaches that make use of wearable sensors. After this, a mathematical model is developed in order to build a link between a number of measures, including blood pressure, skin temperature [18], electroencephalograph readings (which measure the electrical activity of the brain) [19] and heart rate, with the intention of forecasting the thermal sensation of a person. Li et al. designed a smart bracelet device that can detect the wrist skin temperature and its time difference together with the heart rate, which can be used to accurately evaluate the human body’s thermal sensation in different activity states [20]. Reference [21] continuously measured the individual’s electroencephalogram (EEG) signals while wearing an EEG cap, and it used an integrated machine learning method to build a discriminant model to infer the thermal comfort state of the occupants. In light of the fact that the PMV models do not include any uncertainty information, physiological variables continue to be unaffected by the constraints of these models. Such robust representation allows us to obtain more discriminative thermal sensation physiological characteristics of the occupants. Although these methods have achieved promising results, they are limited by user involvement requirements and skin contact requirements. In addition, user acceptance of the sensor configuration is an important factor that affects the promotion and application of a system.

In the realm of computer vision, there are three specific methods for predicting thermal sensations: skin-temperature-based approaches, skin-color-based approaches and posture-estimation-based techniques [22]. The idea that the temperature of the skin may serve as a trustworthy indicator of thermal perception is the foundation upon which the evaluation of skin temperature is built [23,24]. There are a number of studies that have used image processing technologies in order to determine the temperature of the skin in various parts of the body and to then derive the thermal sensation from the skin temperature that was recorded. By comparison to other visible regions of the body, it can be seen that the face is the one that attracts the most attention, particularly the forehead and cheeks, which are the most frequently used areas. Due to the fact that it precisely reflects these feelings, the skin of the face is exceptionally useful for detecting thermal sensations [25,26,27]. Furthermore, scientists have examined the skin temperature of the hand [28,29,30] as well as several other regions of the upper body, such as the elbows and head [31]. In order to provide accurate predictions about thermal comfort, these systems depend on the precision of the skin imaging and detection methods. As a result, deep learning models have been used in research projects that attempt to detect the skin temperature in order to evaluate different parts of the skin. There are several studies that have used OpenPose [32], a computer vision library, in order to accurately recognize elbows and faces in RGB images [33]. Furthermore, in order to determine the temperatures of the faces shown in the thermal images, they used the Haar-based face detector, which is a conventional method of computer vision investigation. However, manually extracting the skin temperature results in a significant decline in the recognition accuracy of an algorithm when dealing with garment insulation and a variable room temperature, which leads to dynamic situations. Moreover, specialized devices, such as thermographic cameras [25,31], are needed to improve the image quality.

Thermal sensation prediction systems use skin pigmentation to extract certain attributes and determine the skin’s temperature. The strategies used in these investigations may be classified as follows. The skin color analysis of the backhand in hand photographs was used to determine the skin temperature in [18,34,35], while the skin color analysis of the face and cheeks was used to evaluate thermoregulation using webcam images in [24,36]. Multiple studies have used a deep learning system to compute the skin temperature by evaluating the hue of the hand’s skin [18,35]. The process of enhancing pre-trained models like Inception and DenseNet201 was realized by using a significant amount of manually captured image data and skin temperature data obtained via the iButton sensor. The Euler video magnification (EVM) approach was used in this study to detect tiny fluctuations in skin color in RGB pictures. The EVM approach improves the detectability of information that is not readily perceptible to the human eye. This methodology was used in all experiments performed in this research [37]. As previously stated, the principles of these approaches depend on reliable skin imaging to predict thermal comfort. Furthermore, the images captured by cameras may be affected by lighting, shadow, color and texture, which all have a significant impact on the thermal comfort inference results.

Another strategy that has gained attention in recent years is the application of comfort postures to infer thermal comfort. This approach involves assessing the thermoregulatory responses related to thermal perception, such as stomping the feet or placing the hands around the neck. Previous studies have shown that engagement in certain activities [38,39] may lead to the anticipation of temperature perception. Usually, these methods adopt the essential anatomical features of an individual’s skeletal system to determine the body’s orientation and assess thermal sensitivity. The research in [38] developed a model that classified 12 distinct comfort positions. This technique relies heavily on precise coordinate position data for crucial joints, which might pose difficulties when using computer cameras. As the precision of evaluating personalized thermal comfort improves, the intrusiveness of the techniques may also rise, hence intensifying the need for human involvement. Specifically, the imaging region must be within the camera’s operational range, posing a difficulty for large and autonomous populations. The positioning of the vital joints is determined by the imaging perspective, the proximity to the object and the behavioral tendencies of the individual. In addition, the computer’s camera is often set up with a restricted field of view, resulting in it only capturing a section of the body. This makes it difficult to obtain a comprehensive and unobstructed image of the whole human body. Furthermore, individuals may not be consistently positioned in front of a computer screen and may sometimes be beyond the camera’s range. To address the limitations of the previously described approaches, a study [40] proposed a thermal adaption behavior detection technique that utilizes spatial–temporal graph convolutional networks (ST-GCNs) [41]. The researchers used OpenPose and spatial–temporal graph convolutional networks to construct a model to identify thermal adaptive behavior. Instead of using RGB images for skeletal tracking, they chose to employ surveillance recordings. However, the empirical evidence suggests that the process of graph generation in ST-GCNs is subject to several restrictions. The skeleton graph used in ST-GCN is pre-determined by heuristic techniques and only captures the physical arrangement of the human body. Hence, this graph does not consistently depict the most optimal choice for action recognition. The interaction between two hands, as shown in activities like ‘clapping’ and ‘reading’, is essential for the recognition of certain categories. Nevertheless, due to the substantial separation of the hands in traditional human-body-oriented networks, ST-GCN has difficulty with precisely capturing their interactions. To improve the responsiveness of the technique, we use a self-attention model called a transformer [42]. This model enhances the sensitivity to tiny behavioral responses.

**Figure 1:**
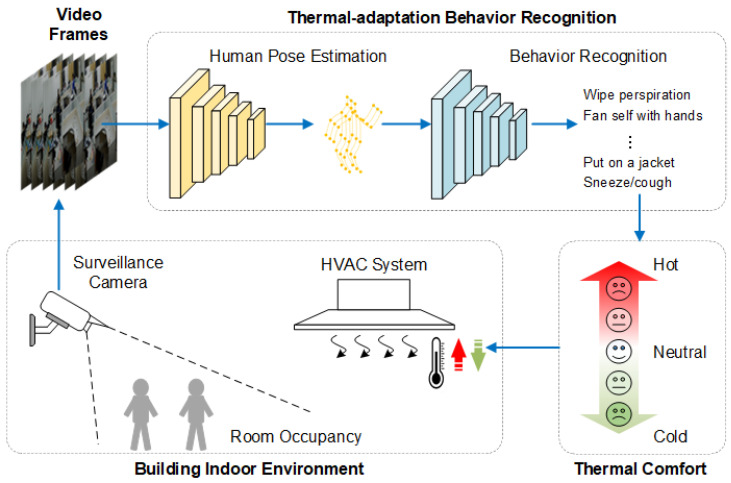
The envisioned framework for occupant-behavior-based HVAC control.

To address the previously stated limitations, we have developed a personalized thermal comfort model that integrates a transformer, which is named the two-stream transformer-enhanced adaptive graph convolutional network (2S-TAGCN). Specifically, the model backbone adopts the graph convolution network model of a deep neural network and integrates transformers to enhance the network performance. The two-stream information of the human body’s bones and joints is simultaneously input into the neural network. Figure 1 shows the envisioned framework for occupant-behavior-based HVAC control. This model employs surveillance cameras to continuously monitor physiological signals, such as temperature adaptation activities, over prolonged durations in real-world settings. The recognition results of thermal adaptation behavior are achieved through two steps: human posture estimation and behavior recognition. Then, the corresponding thermal sensory state is obtained based on the analysis of the occupant’s thermal adaptation behavior. Finally, the thermal sensation is converted into expected temperature regulation signals (such as heating or cooling) and fed back to the indoor HVAC control system to form closed-loop control. Personalized comfort models use customized data rather than aggregated data to predict thermal comfort. This approach allows for the understanding of each occupant’s distinct comfort needs and preferences and supports the delivery of a customized thermal comfort level accordingly. Utilizing personalized comfort models, a building system may create optimal conditions to improve thermal comfort and energy efficiency. Customized models have the capability to adapt and include new data acquired from intelligent buildings, hence allowing the model to evolve and improve. The main contributions of this study are summarized as follows.

- A novel two-stream transformer-enhanced adaptive graph convolutional network for thermal adaptation behavior recognition employing a self-attention mechanism on the spatial graph convolution dimensions is developed.
- A spatial self-attention module based on a transformer is introduced to evaluate the connections and correlations between bone joints in each video frame.
- On thermal adaptation action recognition datasets, the performance of the proposed 2S-TAGCN method significantly exceeds that of state-of-the-art methods.

The subsequent sections of this work are structured in the following manner. A comparison of similar works is presented in Section 2. Within Section 3, a full analysis of the processes and procedures that are covered is provided. Section 4 presents the materials and analyzes the experimental outcomes. Lastly, we provide the discussion and concluding thoughts in Section 5 and Section 6, respectively.

## 2. Related Work

### 2.1. Skeleton-Based Thermal Adaptive Action Recognition

Sensing research is in its early stages at present and focuses on using thermal adaptation behavior to recognize actions by analyzing skeleton-based information. The research in this field may be divided into two categories: manually constructed algorithms that rely on features [38,39] and algorithms that use spatial–temporal graph convolutional networks [40]. Algorithms that depend on manually designed features obtain visual input using computer cameras or Kinect sensor devices. However, these gadgets possess restricted versatility with regard to various scenarios and have a poor level of resilience. Graph convolutional neural network approaches use surveillance cameras to collect behavioral data in a noninvasive way and generate databases of temperature adaptation behavior. This improves the usage of deep convolutional networks in this field. The skeletal data are encoded as a graph structure using a spatial–temporal graph convolutional network. This eliminates the need for manual allocation and traversal requirements, resulting in a faster pace compared to previous techniques. However, the process of generating graphs in ST-GCNs has many limitations. The skeleton graph used in ST-GCN is pre-determined by heuristic techniques and only depicts the physical arrangement of the human body. Hence, there is no guarantee that this graph is the optimal choice for action recognition tasks. An example of the importance of the link between the hand and the head is seen in understanding activities such as ‘head scratch’ and ‘put on a hat’. However, because of the specific positioning of the hands and head in human-body-based graphs, ST-GCN has difficulty with accurately capturing the interconnections among these body components. In addition, the model does not take into account second-order information, such as bone locations and orientation. To address this problem, several studies [43,44] have proposed research initiatives to improve the accuracy of bone-based behavior recognition models.

### 2.2. Transformers in Computer Vision

There have been a number of applications for transformer self-attention modules in the field of computer vision. Some of these applications include video classification [45,46], image convolution [47], image captioning [48], object detection [49], segmentation [50], multimodal tasks [51], generative modeling [52] and action recognition [43]. The capacity of the transformer to properly capture complex interactions in both spatial and temporal dimensions is essential to its excellent achievements in the previously mentioned tasks. On the basis of the input feature, action recognition strategies that make use of transformer self-attention operators may be divided into two primary categories: networks that make use of RGB features and networks that depend on skeleton-based action recognition. Regarding normal image convolutions, the vision transformer (ViT) [46] is a model that makes use of transformers. The fact that it is based on RGB characteristics demonstrates how effective transformers are in this domain. The first effort to apply a transformer model for skeleton-based action recognition used a spatial–temporal graph convolutional network (ST-TR) [43]. The transformer self-attention operator is used in this method in order to illustrate the reliance that exists between joints. This study’s primary purpose is to improve the efficiency of an adaptive graph convolutional network by including a transformer self-attention operator. This will be accomplished by incorporating the operator. Through the acquisition of knowledge about dynamic skeletal information, the objective is to improve the accuracy of recognizing thermal adaption behavior among humans.

The transformer generates new body joint embeddings by comparing pairs of joints and combining their embeddings based on how relevant each joint is to other joints. A self-attention mechanism allows better features to be extracted from each joint by accumulating clues from the surrounding joints, dynamically creating relations within and between human-body-based graphs.

## 3. Transformer-Augmented Adaptive Graph Convolutional Network

The purpose of this study is to increase the accuracy of recognizing thermal adaption behavior by establishing a two-stream adaptive graph convolution network with a transformer. Within this section, we provide a framework for the identification of thermal adaption behavior. This study builds on earlier research in this area. An in-depth discussion of 2S-TAGCN, which is a dual-stream transformer-augmented adaptive graph convolutional network, may be found in this section. The fundamental elements of this framework consist of an adaptive graph convolutional network (AGCN) and a transformer-augmented adaptive graph convolutional network (TA-GCN), which are detailed in Section 3.2 and Section 3.3, correspondingly. The article provides a comprehensive explanation of the transformer self-attention modules as well as AGCN.

### 3.1. Graph Construction

**Figure 2:**
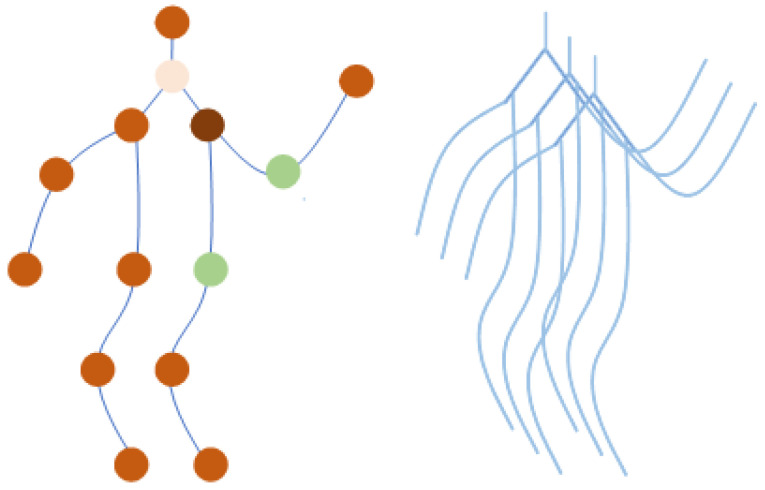
The graph used in the ST-GCN model (**left**) and the bone graph used in the 2S-ACGN model (**right**).

In earlier iterations of skeleton-based action detection challenges, the raw skeletal data were represented as a sequential arrangement of vectors. This was the case in this particular task. Each vector specified the coordinates of the related human joint in either two or three dimensions. An extended motion consisted of a large number of frames, each of which was the duration of a distinct sample. This particular method of combining data is not appropriate for the accurate portrayal of information regarding motion since it is not adequate. An example of a spatiotemporal graph is used by the ST-GCN in order to illustrate the structural information that is shared by these junctions at both the spatial and temporal levels. In addition, a two-stream adaptive graph convolutional network (2S-AGCN) [44] has shown that bone lengths and orientations are often more informative and discriminative with regard to the recognition of activities. With regard to the skeleton dataset, information about the bones has been used as extra data. In light of this, the bone information presented in this research is consistent with the framework that was established by the 2S-AGCN model. A schematic that is referred to as the spatiotemporal skeleton diagram can be seen on the left side of Figure 2. This picture represents the joints as vertices, and the edges represent the spatial connections that exist between the joints inside the human body. A collection of nodes is considered and is denoted as G=(V,E), where $V=\{v_{ti}|t=1,\cdots ,T,i=1,\cdots N\}$ represents the set of all nodes $v_{ti}$ of the graph and $E_{S}=\left\{\left(v_{ti},v_{tj}\right)|i,j=1,\cdots ,N,t=1,\cdots ,T\right\}$ represents the set of all connections between nodes in a frame (the orange lines in Figure 2, left). The temporal edges are denoted as $E_{T}=\left\{\left(v_{ti},v_{(t+1)j}\right)|i,j=1,\cdots ,N,t=1,\cdots ,T\right\}$, while the value of t can range from 1 to *T*. The connection between major articulations in successive phases is shown by these pairs.

Video frames are consecutively arranged along the temporal axis. Afterwards, the coordinate vector of each joint is transferred to the corresponding vertex as an attribute. This process is repeated for each joint. Figure 2 (right) illustrates the bone graph, which depicts the lengths and orientations of bones as a vector that extends from their beginning joint and extends towards their ultimate joint. This vector originates from the first joint of the bone. Let us consider a situation in which we have a bone that is described by an initial joint $v_{1}=\left(x_{1},y_{1},z_{1}\right)$ and a target joint $v_{2}=\left(x_{2},y_{2},z_{2}\right)$; the bone’s vector is $e_{v_{1},v_{2}}=\left(x_{2}-x_{1},y_{2}-y_{1},z_{2}-z_{1}\right)$. Therefore, each bone performs a specific purpose in the body. On the other hand, there is an imbalance in the number of bones and joints since the central joint does not have a bone that corresponds to it. For the purpose of ensuring that the input data are consistent throughout, this approach requires the incorporation of a null bone at the pivotal joint that has a numerical value representing zero. Because of this, the size of the joint is directly proportional to the form of the bone.

### 3.2. Adaptive Graph Convolutional Network

A number of spatiotemporal adaptive graph convolutions are performed on the graph in order to extract high-level properties. The bone and joint graph that was described previously is used in this process. After this, the attributes that were gathered are used in order to apply a global average pooling layer and a softmax classifier in order to make a prediction about the action category. In addition, the topology of the graph, as well as other network characteristics, is optimized via an all-encompassing learning process that stretches from the beginning to the end. A major improvement to the flexibility of the model is brought about by the fact that the graph of each layer and sample is separate. The residual branch ensures the stability of the initial model.

In the spatial dimension, the graph convolution formulation can be summarized as [44]

(1) $$f_{out}=\sum_{k}^{K_{v}}W_{k}f_{in}\left(A_{k}+B_{k}+C_{k}\right)$$

where $f_{out}$ denotes the feature map, which is a C×T×N tensor, where *C* is the number of channels, *T* is the temporal length, and *N* is the number of joints. $K_{v}$ denotes the kernel size of the spatial dimension. Based on the partition strategy designed in [41], $K_{v}$ is set to three. Figure 2 depicts the procedure, with the right image representing it. The green circle serves as a symbol for the centroid of the skeleton. The set of joint points within the region enclosed by the curve can be divided into three subsets: a centripetal subset, which includes neighboring vertices located close to the center of gravity; a centrifugal subset, consisting of neighboring vertices located far from the center of gravity; and the vertex itself. The variable $W_{k}$ represents a weight vector that may be taught and serves as an indicator of the weighting function. $A_{k}$ represents the initial standardized N×N adjacency matrix that illustrates the anatomical structure of the human body. The matrix $B_{k}$ is an N×N adjacency matrix. The acquisition of this matrix takes place throughout the whole training phase, indicating that the graph is exclusively trained using the training data. The matrix components represent both the existence of connections between two joints and the strength of these connections. $C_{k}$ is a graph that dynamically adjusts to the data and creates a unique network for each individual sample. It uses a standardized embedded Gaussian function to determine the correlation between two vertices.

### 3.3. Transformer-Augmented Adaptive Graph Convolutional Layer

The computation of the adaptive graph convolution for the provided skeleton data entails the comparison of adjacent nodes. Nevertheless, this method may not be the most efficient for detecting the heat adaptation behavior of occupants. The categories ‘Scratch one’s head’ and ‘Blow into one’s hand’ require a more profound connection between the hands and the brain, suggesting that the graph structure should be dependent on the data. To address this issue, we propose including a graph convolutional layer that leverages transformer self-attention. This layer has the capacity to independently identify interrelated connections that are crucial for effectively predicting the current action. The kernel values are predicted in a dynamic manner utilizing the identified interconnected relationships, similar to the process of graph convolution. At the sequence level, an identical approach is used to evaluate the alterations to every joint throughout an action and to construct comprehensive linkages that include multiple frames. The resulting operator may create dynamic representations that include spatial dimensions and temporal dimensions.

Moreover, this operator is specifically intended to function as a residual branch, ensuring the stability of the original model. Equation (1) determines the configuration of the adaptive graph based on $A_{k}$, $B_{k}$ and $C_{K}$. $A_{k}$ evaluates the existence of connections between two joints, $B_{k}$ measures the strength of these connections, and $C_{K}$ verifies the presence of linkages between two vertices. To ensure that the graph structure is flexible and includes all global joint interactions, we reformulate Equation (1) as

(2) $$f_{out}=\sum_{k}^{K_{v}}W_{k}f_{in}\left(A_{k}+B_{k}+C_{k}\right)+Z$$

The supplementary element, labeled *Z*, is obtained by computing the correlations between every pair of joints in each frame separately. Within each frame, self-attention is used to extract the low-level information that signifies the interactions between different bodily components. These data are used to ascertain the presence of a correlation between two joints and to measure the magnitude of this association. The approach, also known as the scaled dot-product attention technique, may be defined as follows:

(3) $$z_{i}^{t}=\sum_{j}softmax\left(\frac{\alpha_{ij}^{t}}{\sqrt{d_{k}}}\right)v_{j}^{t}$$

where $z_{i}^{t}\in R^{C_{out}}$ (where $C_{out}$ is the number of output channels) is the new embedding of node $v_{ti}$. We use this method to measure the similarity of two joints in the embedding space. In detail, for each joint (vertex) embedding $w_{i}\in W=\left\{w_{1}\cdots w_{n}\right\}$, a query $q\in R^{d_{q}}$, a key $k\in R^{d_{k}}$ and a value vector $v\in R^{d_{v}}$ are computed independently by trainable linear transformations. Then, a score for each joint embedding is obtained by taking the dot product $\alpha_{ij}=q_{i}\cdot k_{j}^{T},$∀i,j={1,⋯,n}, where *n* is the total number of joints considered. This score indicates how relevant joint *j* is to joint *i*.

**Figure 3:**
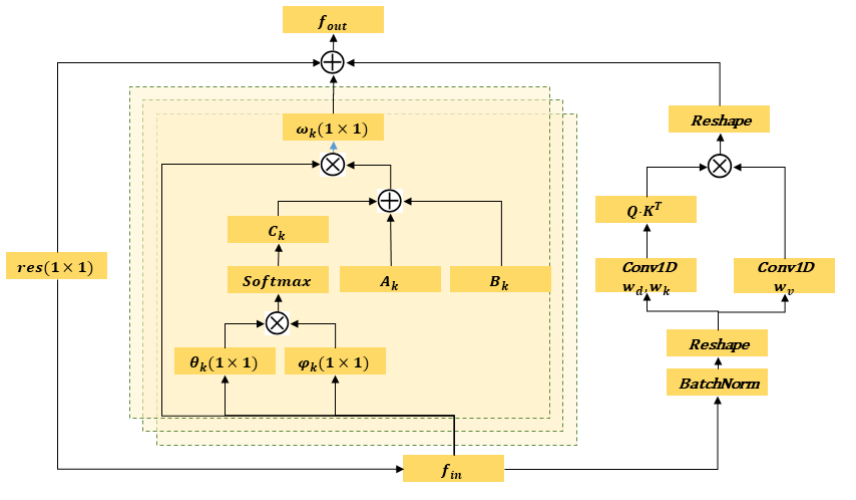
Illustration of a transformer-augmented adaptive graph convolutional layer.

Figure 3 shows the overall architecture of the data flow of the adaptive graph convolution layer. Let $A_{k}$, $B_{k}$ and $C_{k}$ be the variables introduced in Equation (2). The weighting function is denoted as $W_{k}$, with its parameter represented by $W_{k}$. The variable *z* is the similarity measure between bone locations, as stated in Equation (3). The kernel size of the convolution is denoted as (1×1). $K_{v}$ represents the cardinality of the subsets. The symbol ⨁ represents the operation of adding corresponding elements together. The symbol ⨂ represents the operation of matrix multiplication.

**Figure 4:**
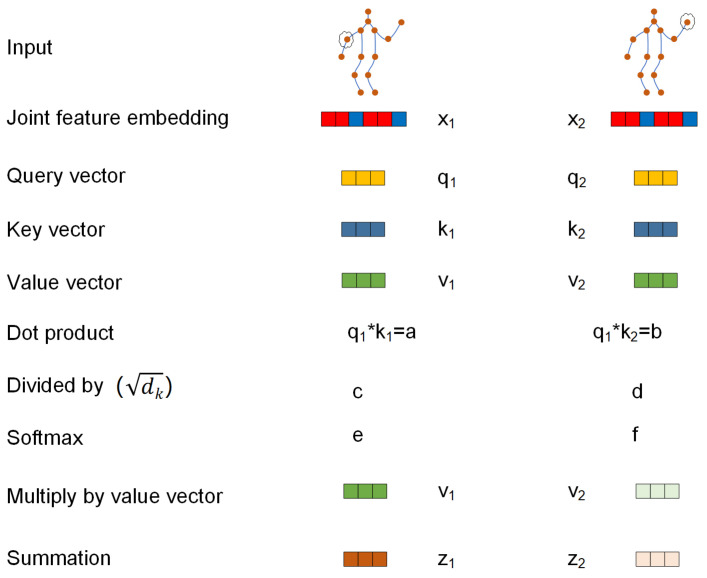
Joint attention calculation based on the transformer.

The interconnections between nodes, represented by the $\alpha_{ij}^{t}$ scores, are dynamically forecast, as seen in Figure 4. Consequently, the correlation structure of the skeleton is not constant throughout all actions, but rather, it is adjusted flexibly for each individual sample. The approach works in a manner similar to a graph convolution on a network that is completely linked. However, in this instance, the values of the kernel (i.e., the $\alpha_{ij}^{t}$ scores) are predicted dynamically based on the skeleton position.

Given the input feature map $f_{in}$ obtained from a frame at time *t* with a size of $C_{in}\times T\times N$, we first reshape the map into $X_{V}$, which has a size of $T\times C_{in}\times N$. The *T* dimension is included inside the batch dimension, enabling the sharing of parameters throughout the temporal dimension while performing distinct modifications on each frame. The technique, referred to as scaled dot-product attention, incorporates a softmax operation and may be computed in matrix form as follows:

(4) $$head_{h}\left(X_{V}\right)=Softmax\left(\frac{\left(X_{V}W_{q}\right){\left(X_{V}W_{k}\right)}^{T}}{\sqrt{d_{k}^{h}}}\right)\left(X_{V}W_{v}\right)$$

where the parameters $W_{q}\in R^{C_{in}\times N_{h}\times d_{q}^{h}}$, $W_{k}\in R^{C_{in}\times N_{h}\times d_{k}^{h}}$ and $W_{v}\in R^{C_{in}\times N_{h}\times d_{v}^{h}}$ are shared across all nodes. Their product yields $Q\in R^{T\times N_{h}\times D_{q}^{h}\times N}$, $K\in R^{T\times N_{h}\times D_{k}^{h}\times N}$ and $V\in R^{T\times N_{h}\times D_{v}^{h}\times N}$. To improve the performance of the algorithm, a process known as multihead attention is commonly used. This process involves applying attention, i.e., a head, numerous times with various learnable parameters and combining the results. The outputs of all heads are then concatenated and projected as follows:

(5) $$Z=SelfAttention_{V}=Concat\left(head_{1},\cdots ,head_{N_{h}}\right)W^{o}$$

where $N_{h}$ is the number of heads and $W^{o}$ is a learnable linear transformation that can be combined with the head output. The embedding extraction technique is iterated $N_{h}$ times, with each iteration using a unique set of trainable parameters, in order to accomplish multihead attention. Subsequently, the output undergoes transformation $R^{\left(C_{out}\times T\times N\right)}$.

Rather than directly replacing $A_{k}$, $B_{k}$ and $C_{k}$ with *Z*, we introduce *Z* into the algorithm. As a result, the flexibility of the model can be improved without sacrificing the initial performance.

### 3.4. Attention-Augmented Adaptive Graph Convolutional Network

The network has units that perform spatial–temporal graph convolution operations. Every unit integrates convolutions in both the spatial and temporal dimensions, as seen in Figure 4. Attention augmentation is used to amplify the spatial dimension at the skeleton level. The output of the spatial GCN is sent via a batch normalization (BN) layer and a rectified linear unit (ReLU) layer. The temporal convolution is performed in the same manner as in the ST-GCN model and uses a kernel size with a $K_{t}\times 1$ format on the C×T×N feature maps.

The output of the temporal graph convolutional network (GCN) is then sent via a batch normalization layer and a rectified linear unit layer. To address the issue of overfitting, we include a dropout layer with a dropout rate of 0.5. As stated before, a residual connection is used to guarantee the stability of the training process.

The attention-augmented adaptive graph convolutional model is composed of nine units in each stream, with channel sizes of 64, 64, 64, 128, 128, 128 and 256. An input data standardization process is implemented via the use of a data BN layer. Ultimately, a global average pooling layer of dimensions is added before the softmax classifier to ensure that feature maps of different sizes from different samples are standardized. After performing these steps, the graph generates feature maps at a higher level, which are then categorized into the appropriate action category using a standard softmax classifier.

### 3.5. Two-Stream Network

**Figure 5:**
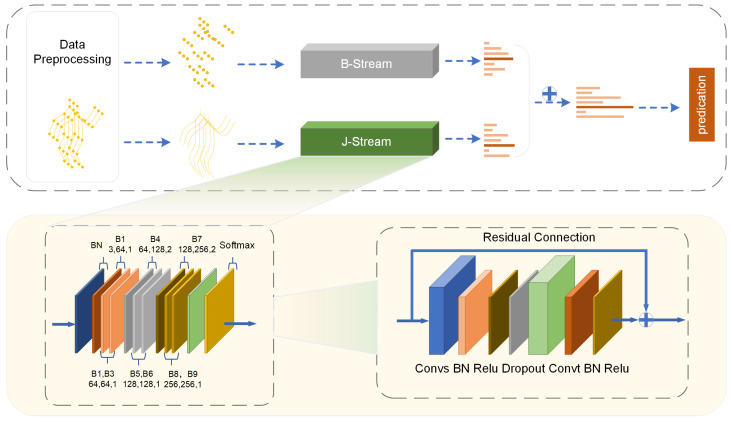
The architecture of the two-stream transformer-augmented adaptive graph convolutional network. ⨁ denotes the element-wise addition.

The two-stream network design is identical to that of the 2S-AGCN. In addition, we use bone data to improve the detection of thermal adaptation actions. As stated in Section 3.1, the graph representing the bones is identical to the graph representing the joints. Therefore, the bone network may be constructed using the joint network as a basis. To differentiate between the joint and bone networks, we utilize J-stream and B-stream, respectively. Figure 5 displays the comprehensive architecture. Specifically, the joint data and bone data are first sent into the matching network to acquire the recognition outcomes. Subsequently, the outcomes are combined with the softmax classifier to ascertain the ultimate behavior label.

## 4. Model Evaluation

Experiments were conducted to ascertain the viability of the proposed methodology. We were specifically interested in ascertaining whether the suggested technique could be used to analyze thermal adaption behavior by comparing the deduced activities at different temperatures as well as whether the deduced states might serve as indicators for the forecasting of thermal comfort.

In this section, the dataset on which the experiments were carried out is first introduced. Section 4.2 shows the parameters and details for network training in the experiment. The final subsection presents the results of the experiments.

### 4.1. Database

A dataset including films from thermal comfort research initiatives was uploaded; we specifically focused on thermal adaptation actions (TAAs) [40]. The dataset had around 14,800 movies that are deemed legitimate and that showcase 16 distinct temperature adaptation behaviors. Specifically, adaptive behaviors for occupants experiencing heat included ‘Fan self with an object’, ‘Fan self with hands’, ‘Fan self with one’s shirt’, ‘Roll up sleeves’, ‘Wipe perspiration’ and ‘Scratch head’, ‘Take off a jacket’, and ‘Take off a hat/cap’. The adaptive behaviors of occupants who felt cold included ‘Sneeze/cough’, ‘Stamp one’s feet’, ‘Rub one’s hands’, ‘Blow into one’s hands’, ‘Cross one’s arms’, ‘Narrow one’s shoulders’, ‘Put on a jacket’, and ‘Put on a hat/cap’. Each video had a length ranging from 5 to 10 s. The movies, captured by Kinect sensors and security cameras, underwent a resizing process, resulting in a resolution of 340×256. Additionally, the frame rate was adjusted to 30 frames per second (FPS). Throughout the training process, we used OpenPose [32] to extract unprocessed skeletal information and construct datasets exclusively composed of skeletal data. Each vector denoted the 2D coordinates of the relevant human joint.

### 4.2. Experimental Settings

All experiments used the PyTorch deep learning framework. The optimization technique used was stochastic gradient descent (SGD) with Nesterov momentum set to 0.9. The total number of batches was 64. The loss function used for the gradient backpropagation algorithm was cross-entropy. The initial learning rate was set to 0.1 and then decreased by a factor of 10 at epochs 60 and 90. These settings were selected based on their demonstrated efficacy for producing favorable outcomes on the ST-GCN network.

We used AGCN modules to adaptively raise the learning rate linearly during the first epoch. In order to mitigate overfitting, we used a dropout technique to regularize the attention weights in the transformer network. This approach entails the random elimination of columns from the attention logit matrix. The multihead attention mechanism was configured with 8 heads in all of the experiments, and the $d_{q}$, $d_{k}$ and $d_{v}$ embedding dimensions were set to $0.25\times C_{out}$ in each layer. A grid search was not applied to these parameters. In terms of model design, each stream included 9 layers with channel dimensions of 64, 64, 64, 128, 128, 128, 256, 256, 256 and 256. Before the softmax classifier was applied, the input coordinates were batch-normalized, a global average pooling layer was applied, and each stream was trained using the conventional cross-entropy loss.

### 4.3. Results

As stated in Section 3.3, the adaptive graph convolutional block has four types of graphs: A, B, C and T. We manually excluded one of the graphs and present the resulting performance in Table 1. This table demonstrates the usefulness of adaptively acquiring knowledge about the graph for action recognition. It also highlights the negative impact on performance when any one of the three graphs is removed. Another notable advancement is the use of second-order data. This section provides a performance comparison between Js-AGCN and Bs-AGCN for each of the input data types, as shown in Table 2. The results also illustrate the influence of using various types of input data in the 2S-AGCN model.

Another significant advantage is the use of second-order information. In this section, we evaluate the performance of various input data formats (Js-AGCN and Bs-AGCN in Table 2) and compare it to the performance achieved when the data types are combined (represented by 2S-AGCN in Table 2), as described in Section 3.5. The use of the two-stream strategy yields superior results compared to the one-stream solutions.

When compared with the ST-GCN model, the 2S-GCN model shows significant advantages for most thermal adaptation behaviors, especially those with smaller motion ranges, such as shoulder rolling, arm holding, stomping and head scratching, as shown in Table 3. In addition, the ST-GCN model has the lowest accuracy when identifying heat and cold discomfort behaviors of sweat wiping and shoulder pulling. The performance of these two behaviors reaches 70.21% and 71.28%, respectively, and the 2S-TAGCN model improves the recognition accuracy of these two behaviors to 72.74% and 93.62%, respectively.

**Figure 6:**
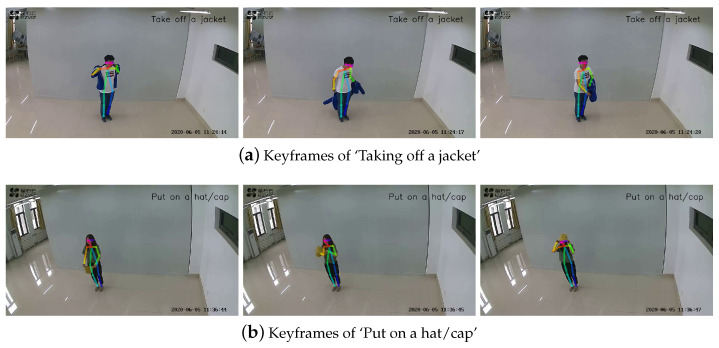
Visualization of partial thermal adaptation behavior recognition results.

The precision of sweat wiping was only 72%. However, the experimental results suggest that the ST-GCN model did not achieve a high level of accuracy at identifying this behavior. This can be attributed to the intricate nature of the movement and the substantial diversity of behavior among individuals. Figure 6 displays a visual depiction of the recognition outcomes of the proposed method for different thermal adaptation behaviors in surveillance videos. Figure 6a presents the key frames that depict the action of ‘Taking off a jacket’, while Figure 6b showcases the key frames that illustrate the action of ‘Put on a hat/cap’.

The precision of thermal sensations was assessed using the model established in our prior investigation. The probabilities of thermal comfort predictions $P_{T}$ were computed using the equation. The user’s text consists of the number (6) enclosed in parentheses:

(6) $$P_{T}=P\left(C|A_{i}\right)\times P_{accuracy}$$

where $P_{accuracy}$ is the behavior recognition accuracy, which is in line with previous work.

The precision of forecasting thermal perception is contingent upon the precision of behavior recognition quantified and the probability of a certain behavioral action being linked to thermal sensation. The probability of a certain behavioral reaction linked to the perception of temperature is consistent. However, the accuracy of anticipating thermal sensation varies depending on the precision of behavior recognition. The experimental results indicate a substantial improvement in the accuracy of identifying behavior, resulting in a comparable increase in the accuracy of prediction.

## 5. Discussion

The data presented in Table 2 show that the average accuracy of the proposed method at identifying 16 thermal adaptation behaviors reaches 96.56%. This means that the occupant data acquired by the surveillance cameras as sensors can be used to effectively analyze and infer the thermal adaptation behavior of the occupants in the room. This model realizes the process of non-invasive identification of thermal adaptation behavior without the need to install special equipment in the scene, and the occupants do not need to wear detection equipment.

The aim of this study was to identify 16 thermal adaptation behaviors related to occupants’ thermal comfort. Table 3 shows the improvements to the accuracy of each behavior’s recognition compared with the ST-GCN method. The thermal adaptation behavior with the lowest recognition accuracy is ‘Wipe perspiration’, which is only 72.74%. The highest behavior recognition is reflected in ‘Take off a jacket’ and ‘Cross one’s arms’, where it reaches more than 99%. This reflects the differences in the sensitivity of the proposed methods to different actions. Future work will consider the need to design more appropriate strategies for the classification of specific thermal sensory behaviors. On the other hand, the scale and diversity of data can improve the generalization capabilities of the model. Therefore, in our next work, we will consider exploring more visual behaviors related to human thermal sensation while increasing the size and diversity of the dataset.

When using cameras to map occupants’ minute movements, it is worth considering potential privacy violations. The main aim of the study was to explore a possible method of optimizing thermal sensitivity and reducing energy use that could be deployed in private spaces with the permission of the occupants. In order to avoid privacy issues as much as possible, the method proposed in this article only infers information based on the skeletal characteristics of indoor occupants, and it does not collect private information such as the appearance, clothing, gender, etc. of the occupants through cameras. This means that the model only focuses on the skeletal motion that occurs and is not associated with the object on which the motion occurs. Finally, the final output of the model is only the thermal sensation, which is directly converted into input information for the temperature control module of the HVAC system in subsequent applications. Therefore, the identification process does not involve any personal information.

The main limitation of this study is that, although it achieved high accuracy at identifying 16 types of thermal adaptation behaviors, the model has a huge number of parameters and high complexity. In future work, further pruning and optimization of the model will be considered to enable the solution to be integrated into existing terminal equipment for the inference of thermal comfort behavior. Integrating the thermal comfort identification algorithm into the existing monitoring equipment in a building can effectively improve the thermal comfort of the personnel in the building while dynamically regulating the air-conditioning system to reduce the building’s energy consumption.

## 6. Conclusions

The accurate inference of the thermal comfort of building occupants can enable buildings to dynamically manage HVAC systems to save energy and reduce emissions. Based on building indoor surveillance camera data, this paper proposes a non-contact method of inferring the thermal comfort of the occupants in a building, which is expected to minimize energy consumption while ensuring the comfort of the occupants. The skeletal data in this model are organized in a graph structure, which is parameterized and incorporated into a network that can be both taught and updated. The model integrates both primary and secondary data from bone measurements, emphasizing the importance of the bone orientation and length in motion detection models based on bone characteristics. The model underwent a comprehensive evaluation of its ability to respond to heat, with a specific focus on the thermal adaptation behavior dataset, and the validity of the model in recognizing thermal adaptation behaviors was verified.

The main contributions of this article include the following: (1) An adaptive graph convolutional network based on a transformer that can adapt to different action recognition tasks and thermal adaptation behaviors by learning the topological structure of graphs in different GCN layers and skeleton samples in an end-to-end manner is suggested. (2) The second-order information from the skeleton data is articulated and coupled with the first-order information, considerably improving the recognition performance. (3) When compared with existing models, the accuracy of the 2S-TAGCN model for recognizing skeleton-based thermal adaptation behaviors in large-scale datasets is greatly improved.

By employing indoor monitoring equipment and deep learning algorithms to identify the thermal adaptation behavior of occupants, we can effectively monitor and predict their thermal comfort, thereby enabling intelligent adjustments to the air-conditioning system’s operating status. This approach not only offers the potential to reduce buildings’ energy consumption but also enhances the personalized thermal comfort of occupants within the indoor environment.

## Figures and Tables

**Table 1:** Comparisons of the validation accuracy when adding adaptive graph convolutional blocks with or without A, B and C; wo/X indicates that the X module was deleted.

Method	Accuracy (%)
ST-GCN	87.66%
TA-GCN wo/A	79.29%
TA-GCN wo/B	73.81%
TA-GCN wo/C	85.79%
TA-GCN wo/T	90.94%
TA-GCN	93.96%

**Table 2:** Comparisons of the validation accuracy with different input modalities.

Method	Accuracy (%)
Js-TAGCN	93.96%
Bs-TAGCN	93.15%
2S-TAGCN	96.56%

**Table 3:** Validation accuracy comparisons of 16 thermal adaptation actions.

No.	Action	ST-GCN	2S-TAGCN
1	Fan self with an object	93.75%	81.25%
2	Fan self with hands	85.42%	79.71%
3	Fan self with one’s shirt	89.58%	87.50%
4	Roll up sleeves	95.83%	93.75%
5	Wipe perspiration	70.21%	72.74%
6	Scratch head	81.25%	93.75%
7	Take off a jacket	98.67%	99.65%
8	Take off a hat/cap	98.85%	97.92%
9	Sneeze/cough	97.87%	97.92%
10	Stamp one’s feet	85.42%	98.96%
11	Rub one’s hands	89.36%	85.11%
12	Blow into one’s hands	98.92%	95.83%
13	Cross one’s arms	93.75%	99.23%
14	Narrow one’s shoulders	71.28%	93.62%
15	Put on a jacket	97.87%	85.42%
16	Put on a hat/cap	98.74%	97.87%

## Data Availability

The data has not been made public due to privacy restrictions. Further inquiries can be directed to the corresponding author.

## Nomenclature

⨁	matrix addition
⨂	matrix multiplication
*G*	the graph representation of the human body
*V*	the set of all nodes
$E_{S}$	connections between nodes
$E_{T}$	temporal edges
*C*	the number of channels
*T*	the total number of nodes
$A_{k}$	joint connection matrix
$B_{k}$	connection strength matrix
$C_{k}$	vertices connection matrix
*Z*	the correlations between joints
$N_{h}$	the number of heads
$W^{o}$	the learnable linear transformation
$W_{k}$	the weight matrix
$K_{v}$	the kernel size of the spatial dimension
$K_{t}$	the kernel size of the temporal dimension
$C_{in}$	the number of input channels
$C_{out}$	the number of output channels
$f_{in}$	input feature map
$f_{out}$	output feature map
ReLU	rectified linear unit
ST-GCN	spatial–temporal graph convolutional network
AGCN	adaptive graph convolutional network
2S-AGCN	two-stream adaptive graph convolutional network
2S-TAGCN	two-stream transformer-enhanced adaptive graph convolutional network
TA-GCN	transformer-augmented adaptive graph convolutional network
TAAs	thermal adaptation actions
FPS	frames per second
BN	batch normalization
SGD	stochastic gradient descent
J-stream	the joint network
B-stream	the bone network
